# Insights about the Epidemiology of Dog Bites in a Canadian City Using a Dog Aggression Scale and Administrative Data

**DOI:** 10.3390/ani9060324

**Published:** 2019-06-06

**Authors:** Niamh Caffrey, Melanie Rock, Olivia Schmidtz, Doug Anderson, Melissa Parkinson, Sylvia L. Checkley

**Affiliations:** 1 Department of Ecosystem and Public Health, Faculty of Veterinary Medicine, University of Calgary, 2nd Floor, TRW Building, 3280 Hospital Drive NW, Calgary, AB T2N 4Z6, Canada; mrock@ucalgary.ca (M.R.); ocschmid@ucalgary.ca (O.S.); slcheckl@ucalgary.ca (S.L.C.); 2 Department of Community Health Sciences, Cumming School of Medicine, University of Calgary, 3rd Floor, TRW Building, 3280 Hospital Drive NW, Calgary, AB T2N 4Z6, Canada; 3 O’Brien Institute for Public Health, University of Calgary, 3rd Floor, TRW Building, 3280 Hospital Drive NW, Calgary, AB T2N 4Z6, Canada; 4 City of Calgary, Calgary Community Standards, 2201 Portland St SE, Calgary, AB T2G 4M7, Canada; tjej@telus.net (D.A.); melissa.parkinson@calgary.ca (M.P.); 5 Provincial Laboratory for Public Health, Alberta Public Laboratories, Alberta Health Services, Calgary, AB T2N 4W4, Canada

**Keywords:** animal welfare, bite prevention, dogs, dog bite, Dunbar scale, dog aggression, One Health, wounds and injuries

## Abstract

**Simple Summary:**

Any dog is capable of biting, and dog bites can cause serious injuries to humans or other animals. To prevent dogs from biting, we need to understand the circumstances in which dog bites are most likely to occur. Once we have that information, we can act by improving public awareness and tailoring interventions to those most at risk of being injured. In this study, we assess the circumstances surrounding dog-bite injuries, by considering dog-bite severity in conjunction with information about where the incident occurred, about human victims, and about the dogs themselves. This approach was possible because The City of Calgary systematically tracks dog-bite severity. We found that from 2012–2017, the number of severe bites occurring in Calgary has fallen. That said, severe bites tended to occur in the homes where the dogs lived, and to be directed towards children and older adults. The results from our study underscore that educational communication for parents, grandparents, and other caregivers should emphasize the importance of constant supervision around dogs, including family dogs, whenever children are present. In addition, more attention should be paid to the risks that dogs pose to older adults. Failure to protect people from dog-bite injuries has implications for the dogs’ own welfare, as well as for human health. Dogs are most likely to bite when they feel insecure in the first place. Furthermore, common responses to severe dog-bite injuries in people include rehoming and euthanizing dogs.

**Abstract:**

Dog bites are a public health concern that also implicates animal welfare, with negative outcomes such as rehoming or euthanasia for the animals responsible. Previous research has shown that the severity of dog-bite injuries reflects multiple factors, including the degree of inhibition exhibited by dogs and how people behave towards dogs. This study utilizes an objective dog bite injury assessment tool: The Dunbar aggression scale. Trained officers employed by The City of Calgary systematically use the Dunbar scale whenever investigating dog-bite complaints. We analyzed The City of Calgary’s administrative data on confirmed dog-bite injuries in people, 2012–2017, with a multivariable generalized ordered logistic regression model. Severe dog-bite injuries occurred more frequently in the family home than in any other setting. Young children, youths and older adults were at higher risk of more serious bites than adults. There has been a decreasing trend in the probability of a high or medium severity bite, and an increasing trend in the probability of a low severity bite since 2012. These results indicate that greater public awareness regarding dog-bite injuries is needed. Consideration should be given to campaigns targeted towards different demographics, including older adults, to provide an understanding of dog behaviour and to emphasize the need to supervise children closely in the presence of all dogs at all times, including family dogs in the home environment. Given that dog-bite injuries are not just a public health issue, but also an animal welfare issue, we endorse One Health responses in educational campaigns, policy development, and professional practice.

## 1. Introduction

Dogs are a common and valued household pet in many countries [1,2,3]. Beyond their innate value as a part of the family unit, canine companions can support increased social engagement and activity levels for owners, especially via dog-walking [3,4,5,6]. Hence people’s relationships with canines have the capacity to promote both mental and physical health, in both the people and the dogs. These benefits, however, do not occur without some risks, including infections and dog-bite injuries. Any dog can potentially bite. It is whether that dog has developed a reliable bite inhibition that drives the extent of physical harm that can occur from the bite. Dog-bite injuries can vary in severity from lacerations, tears, and crush injuries. Bacterial contamination at the site is also a potential health risk [7].

Understanding the factors that lead to instances of dog aggression is paramount when considering methods by which to reduce the health impacts of the problem. There are numerous factors to consider including both human and canine demographics, canine behaviour, and individual circumstances of the incident, such as where it occurred, and familiarity between the dog and victim [8]. The circumstances in which a dog displays aggression can inform experienced and knowledgeable handlers as to the disposition of the animal. In 256 dog bite related fatalities in the USA between 2000–2009, the following potentially preventable factors were considered to have played a role: Owner mismanagement of dogs (37.5%), owners with a history of abuse or neglect of dogs (21.1%), and dogs kept isolated from regular positive human interactions (76.2%) [9]. Animal-related factors such as age, spay/neuter status and breed of the dog have long been implicated as risk factors for dog bites, however, there are no standardized methods for measuring and reporting these data. Furthermore, attempts in the literature to quantify and understand dog behaviours such as growling, snarling, snapping and biting has resulted in confusing and conflicting messages. Aggression is a common reason for relinquishment of dogs to animal shelters, and aggression was a factor leading to a decision for euthanasia in 95% of Canadian animal shelters responding to a survey on euthanasia practices [10]. Therefore, there are significant negative outcomes associated with the exhibition of aggressive behaviour for the animals in question. These factors are indicative of animal welfare concerns that could be addressed through a greater understanding of the risk factors for dog bites in our society. An understanding of this public health issue requires a One Health approach, where we consider the health and welfare of the animals, look to understand the reasons why dogs bite, and we educate ourselves in how to interpret dog language with a view to ultimately curtailing a preventable public health risk.

Research and surveillance on dog bites have historically focused on zoonotic disease transmission [11], with the disease of most concern being rabies. Rabies remains a public health concern, yet dog-bite injuries are much more common than rabies or any other zoonotic infection [12]. The World Health Organization (WHO) laments the lack of global dog-bite surveillance, even as dog-bites surely account for “tens of millions of injuries annually,” with children at the highest risk for dog-bite injuries and fatalities [13]. Given the lack of global surveillance for dog-bite injuries and fatalities, the WHO cites research from the United States to convey the magnitude of the problem. In the United States, survey research has found that dog bites affect approximately 1.5% of the human population every year, totaling more than 4.5 million incidents and 20–40 deaths annually in that country alone. Additionally, as noted by the WHO, countries such as “Australia, Canada and France have comparable incidence and fatality rates.” [13] Data from middle-income and lower-income countries are “more fragmented” [13] than in higher-income countries, where surveillance remains incomplete [14]. In the American city of Pittsburgh, for example, a landmark study found inconsistency in dog-bite reports collected by healthcare, law enforcement, and animal-focused agencies [15].

In Canada, meanwhile, dog-bite surveillance and policy remain fragmented [16]. Under the auspices of the Canadian Hospitals Injury Reporting and Prevention Program (CHIRPP) [17], data are collected routinely in a series of emergency departments that serve as sentinel sites. Analyses of dog-bite data in CHIRPP, however, have not been released since 2005. Nonetheless, the 2005 CHIRRP report suggests that dog-bites remain a top-10 cause of injury in children between 5 and 9 years of age [18]. Policy responses to dog-bite injuries vary across Canada, not least because considerable discretion has been granted to local governments and animal welfare organizations, which differ in their approaches and resource capacity. Breed-specific legislation (BSL) exists in several Canadian jurisdictions, while in other places in Canada, BSL has only recently been repealed or has recently been proposed. In Canada, dog bite prevention educational resources are provided at the local [19] and provincial level [20], as well as through national organizations such as the Canada Safety Council [21].

In this article, we report on quantitative research that was conducted collaboratively between the University of Calgary and The City of Calgary, which has never had BSL. Indeed, The City of Calgary has earned an international reputation for simultaneously reducing per capita dog-aggression incidents and euthanasia of dogs, without recourse to BSL [22,23,24,25]. Calgary’s Responsible Pet Ownership Bylaw aims to reduce bites in urban centers by means of effective licensing, reporting, and ticketing programs. This approach increases revenue to stream into education seminars in schools and encourages veterinary visits [26]. Whereas licensing rates remain low in many Canadian cities [26], Calgary has a high rate of licensure, therefore allowing for better estimates on the dog population and management strategies [3,4,5,27].

Our study uses the administrative data collected by The City of Calgary to analyze dog-bite cases from the period 2012–2017, with the Dunbar aggression scale serving as the outcome of interest. An epidemiological approach to understanding the risk factors associated with varying dog bite severity was undertaken. Previous research into dog bite-related issues has utilized data collected through surveys [28,29,30] or from medical databases [14,31,32]. Additionally, few studies have examined dog bite data using a multivariable approach. Therefore, the use of the Dunbar scale as an outcome of interest in an epidemiological analysis of dog bite data provided by a city administration is novel. The objective of this work is to examine associations between risk factors for dog bites collected by animal services staff, and the severity of the actual bite inflicted, as measured using the Dunbar scale. Risk factors to be evaluated include dog related factors, such as the age, sex, and breed of the dog, and incident related factors, such as the age and gender of the victim, where the incident occurred, geography, who was controlling the animal, and the relationship between the victim of the bite and the dog. Undertaking this analysis will allow for an understanding of factors influencing the severity of a dog bite, and where such incidents occur amongst Calgary’s biting dog population. This will allow for informed decision making related to local policy and intervention strategies. At the same time, given Calgary’s status as a leader in humane animal control policies and procedures, this analysis has international significance.

## 2. Materials and Methods

### 2.1. Data Collection

The City of Calgary-Calgary Community Standards (Animal Services) uses a customizable software known as Chameleon© to track dog-aggression complaints and investigations. The city provided 2012–2017 routine statistics collected upon an incident of dog aggression or dog bite reported to animal services. Reports may come from multiple avenues but predominantly come from citizens. All reported incidents are followed up but only confirmed incidents are reported in the statistics. A confirmed incident is one where evidence has been provided to substantiate the incident. Evidence may not be included when: The victim changes their mind about report, statements are not completed, or the victim never calls the officer back. These incidents are recorded in Chameleon, but they are closed as unfounded. Over the six-year time period from which statistics are available, there were 4433 reported incidents of dog aggression towards people or other animals, of which 2906 (65%) were confirmed.

The Chameleon software provides officers with drop-down menu choices to record information regarding the complaint. When an incident is reported, attending officers collect the following information about the dog in question: The primary breed, age, sex, spay-neuter status, vaccination status, years owned, the place from which the dog was obtained, and whether the dog had previously exhibited aggressive behaviour. The officer collects data about the victim, including the gender, age, and relationship to the dog. The location of the incident, who was in control of the dog at the time of the incident, and the circumstances are recorded. As an example, the drop-down menu choices for recording the circumstances of the incident include the following options: At large, contained, dog fight, guard dog, multi-dog, on a leash, other, provoked, tethered or unknown. There were 153 confirmed incidents that involved multiple dogs. The drop-down menu for the location of the incident includes the following options: In house, off-leash park, owner’s property, or public. Where more than one circumstance might apply in a particular scenario, officers are trained to choose the circumstance that is most pertinent to the situation. For example, if the officers were responding to a call in a public place where a number of uncontrolled dogs were involved in a fight, the officer would record the incident as a ‘dog fight’ as opposed to ‘at large’.

The City of Calgary systematically applies the Dunbar scale when investigating dog-aggression incidents. This is an objective, systematic assessment tool for measuring aggression in dogs [33], modified with the inclusion of useful graphics to accompany interpretation of the scale [34]. Depending on the amount of injury caused, the bite is categorized to one of six levels (Table 1) [35]. A level 1 (pre-bite) occurs when a dog snaps or air bites, but makes no contact with a person or another animal [34]. Such behaviour indicates that the dog was anxious or fearful in the moments preceding the incident. A level 2 incident represents a near-bite or a highly inhibited bite. At this level, a dog will snap and make tooth contact without puncturing the skin [34]. It can be preceded by lunging or charging behaviour. The force of the bite is inhibited at this level. However, bruising may occur at the point of contact. At level 3 on the assessment scale, a dog bites once and punctures the skin with one to three holes [33], with the puncture shallower than the length of the canine tooth [34]. At level 3 there is no tearing or slashes, and the victim has not been shaken from side to side [33]. At level 3.5, the dog bites multiple times at the severity of a level 3 bite [33]. At this level, the dog is highly aroused and reacting without thinking between bites [34]. A level 4 bite consists of two to four holes from a single bite [33]. The punctures are deeper than the length of a canine, indicating that the dog bites and clamps down [34]. The bite may also produce slashes in both directions indicating that the dog shook its head [34]. A level 4 bite indicates that the dog has not shown any inhibition in bite strength, therefore a dog that bites at this level could be considered a dangerous animal. A level 5 bite occurs when the dog gives multiple level 4 bites [33] with deep punctures [34]. Dunbar describes a level 5 bite as “a concerted, repeated attack causing severe injury” [33]. The aggression scale is completed with level 6, which is any bite resulting in the death of a human or another animal [33]. The City of Calgary collate their dog bite incidents into three spreadsheets. The first is ‘Chases’, being all level one or level 2 incidents. The second is ‘Bites’ being all level 3, 3.5, 4 and 5 bites. The third spreadsheet represents dog on other animal aggression incidents. These include incidents recorded at all levels of the Dunbar scale.

### 2.2. Statistical Analyses

Data on chases (levels 1 and 2 on the bite scale) and bites (levels 3–5 on the bite scale) directed towards humans, provided by the City of Calgary in Excel format were merged into a single spreadsheet and imported into Stata IC v15 (StataCorp, College Station, TX, USA) for all data manipulations. The outcome of interest was a three-level ordinal variable indicating if the bite was a Low (level 1 or 2), Medium (level 3), or High (level 3.5, 4, or 5) severity incident (Table 1). It was hypothesized that the severity of a dog bite may be associated with the setting in which the incident took place. For example, it was considered that children would be subject to more severe bites than older persons. It was also considered that more severe bites would occur in the home. It was hypothesized that intact male dogs would be responsible for more severe bites than neutered males or females. The authors considered that the breed of dog was not likely to be associated with bite severity.

The distribution of all variables included in the City of Calgary data was evaluated. The frequency and percentage for categories of each variable along with the frequency of incidents at the three outcome levels of interest are presented (Table 2). All variables were categorical with two or more categories in each variable. When examining categorical variables, each level of the variable was required to include a minimum of 5% of the observations in order to be considered for further analysis. The distribution of many of the variables was skewed, making it infeasible to consider further analytical statistics. For example, the variable ‘Circumstances’ had 10 different categories to describe the circumstances and these categories did not lend themselves to grouping. This was similar for the relationship of the victim to the dog, and who was controlling the dog at the time of the incident.

There were 157 unique entries for the dog primary breed information. Due to the subjectivity involved in classifying dog breed [36], and the huge variation in the types of breed reported, this variable was not assessed. The primary breed group, based on the Canadian Kennel Club classifications [37] classified dogs into one of seven breed group categories. Breed group was assessed in an unconditional model as a predictor of interest despite one category (hounds) making up just 2% of the observations.

There were a number of variables for which data were not collected in every year, therefore there were a large number of responses where the category is listed as ‘unknown’. This included where the animal was obtained, the number of years the owner had the dog, licensing status and vaccination status. For this reason, these variables were omitted from further analysis. There were four observations where victim gender was not recorded, four where the breed group was not specified, and two locations that were listed as ‘private’ that were all excluded from the analysis.

The City of Calgary has 14 electoral ward districts, with the distribution of bites in each ward ranging from 4% to 10%. Information on the ward was unavailable for 14% of incidents. The City of Calgary is also divided into four quadrants. Each ward was placed within the appropriate quadrant for further analysis.

Unconditional associations between the outcome and the variables collected by animal services were explored using Generalized Ordered Logistic regression using the gologit2 package in Stata 15 in order to allow for non-parallel lines assumptions in the modelling process [38]. Variables that had significant unconditional associations (*p* < 0.2) with the outcome that were considered for model building were year, breed group, incident location, the age of the victim, sex of the dog, age of the dog, whether the dog had displayed any prior aggression, and the month of the year (Table 2).

For the final model building process, 548 observations where the age of the victim, the sex of the dog, or the age of the dog were unknown were removed from the analysis. This allowed for a more parsimonious fitting model that accounted for the same level of variation as a model fitted using the same predictors and including those observations where responses were unknown. Victim age was also recategorised so that all 20–59 year olds were in one category (n = 1662) instead of 4 categories (20–29, n = 314, 30–39, n = 511, 40–49, n = 442, 50–59, n = 395). This was done to allow for a more simplified interpretation of the effect of age in the multivariable model.

Predictive margins were used to calculate the predicted probability of the different outcomes for each predictor, with a Bonferroni correction applied to each calculation to account for multiple comparisons. Margins predict the average probability of the outcome if every incident for the predictor of interest were treated as observed and leaving all other predictors as they are. For example, when computing margins for the breed group, margins will predict the probability for herding dogs by treating all incidents as herding dogs and keeping the other predictors at their values. It will then compute the margins for hounds by treating all incidents as hounds, etc. For this reason, the predictive margins calculated do not reflect specifically the model results indicated in Table 3, where predictors are reported with odds ratios (OR) and are considered in relation to a baseline outcome, and controlling for the other predictors in the model.

Pairwise comparisons were also calculated for each predictor to indicate if the differences between groups were statistically significant. The predictive probability calculated for each predictor is presented graphically using Stata margins command and the user-written commands in the SPost13 package [39]. Error bars depict the confidence intervals, which can be used to detect significant differences. Where the confidence interval depicted does not portray the result of a pairwise comparison, this is indicated in the text.

## 3. Results

There were 2723 incidents of chases or bites against humans recorded from 2012 to 2017 of which 2165 were analyzed in the final statistical model. The 2165 incidents involved 1873 dogs, of which 54 dogs (3%) were subsequently euthanized. In this study, 51% of dog aggression incidents directed towards humans reported to the City of Calgary Animal Services between 2012–2017 were classified as level 1 or 2 (low severity) on the Dunbar aggression scale. Thirty-five percent of incidents were classified as level 3 (medium severity), and 13.5 percent were classified as level 3.5 or higher (high severity).

### 3.1. Multivariable Generalized Ordered Logistic Regression Model

A multivariable generalized ordered logistic regression model that included the variables for year, breed group, incident location, the age of the victim, the sex of the dog and the age of the dog was fit using a manual backward elimination strategy (Table 3). This model included 2165 incidents between 2012 and 2017. The autofit option using a conservative significance level of 0.005 was used to test whether variables met the parallel lines assumption. The gamma option was used to report the deviations from proportionality where non-parallel lines were fit [40].

The odds ratios presented represent the change in odds of observing a value above the listed severity level versus observing values at or below the listed severity level [39]. The values in Table 3 represent the odds ratios for incidents that were classified as low, compared to medium and high severity incidents (Equation (1)), and incidents that were classified as low or medium compared to high severity incidents (Equation (2)). Odds ratios for Equation (2) are only reported where they differed from that in Equation (1) (non-parallel lines). The difference in proportionality (non-parallel lines) is assessed by the Gamma *p*-value. For example, the odds ratios for the terrier breed group can be interpreted as follows. Holding all other predictors constant, dogs in the terrier breed group have a 0.71 times lower odds of being responsible for a medium or high severity incident than do dogs from the non-sporting breed group. Terrier breed group have a 1.10 times higher odds of being responsible for a high severity incident than the non-sporting breed group. The difference in proportionality was statistically significant (Gamma *p*-value = 0.003). The odds of an incident occurring in the home was 8.17 times higher than in public places. The same odds ratio was reported for all incident severity levels.

The constant odds ratio 0.40 for the low versus medium and high severity incident represent the odds of a bite in the year 2017 inflicted by an intact female dog, 0–2 years old from the non-sporting breed group, where the injury was inflicted upon an adult in a public space. This odds ratio can be compared to the odds ratio of 0.05 if the bite was in a high versus medium or low severity incident. The average predicted the probability of a low, medium and high severity incident was 0.507, 0.354 and 0.139 respectively.

**Figure 1:**
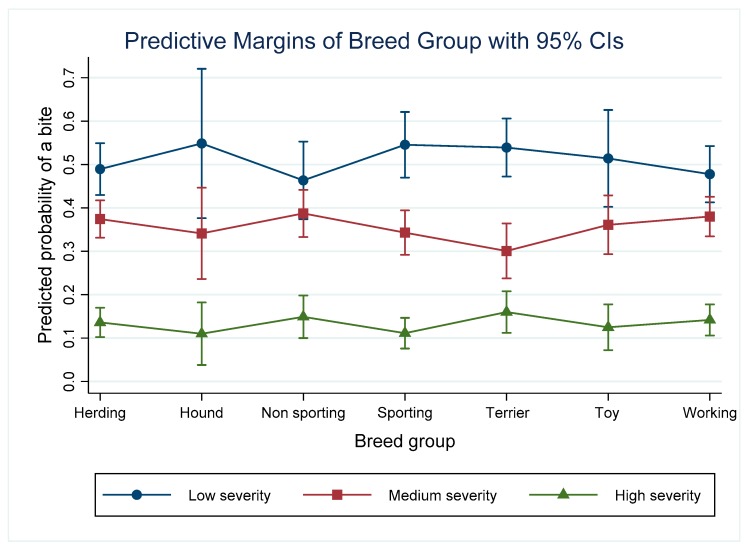
Predictive margins for the different severity incidents depending on the breed grouping and controlling for other variables in the model.

The predictive probabilities depicted in the graphs that follow can be considered in terms of percentages. For example, predictive probabilities of 0.49 for a low severity incident, 0.37 for a medium severity incident and 0.14 for a high severity incident in herding dogs (Figure 1) indicates that there is a 49% chance of a low severity incident, 37% chance of a medium severity incident and 14% chance of a high severity incident in herding dogs, with all other predictors being held constant.

### 3.2. Breed Group

The model depicted in Table 3 indicates that the sporting and terrier breed group had reduced odds of a low versus medium and high severity incident compared with the non-sporting group when all other predictors are held at the baseline as described above. Based on the predicted probabilities of incidents at each severity level, no one breed group stands out as being responsible for a higher percentage of bites. The predicted probabilities of a high severity incident were significantly lower than that for medium and low severity incidents in all breed groups (Figure 1). In the sporting and terrier breed groups, the predicted probability of a low severity incident was significantly higher than a medium severity incident. In the other groups, the differences between the probability of low severity or medium severity incident were not statistically significantly different.

### 3.3. Incident Location

**Figure 2:**
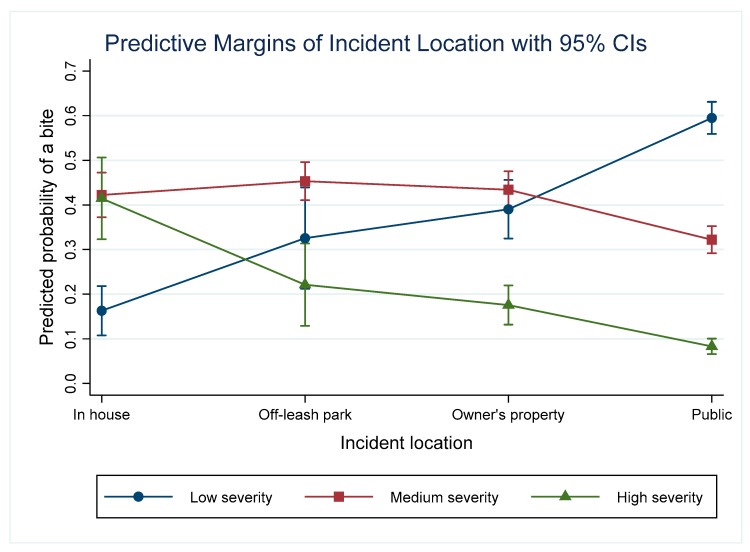
Predictive margins for the different severity incidents depending on the incident location and controlling for other variables in the model.

The model indicates the odds of medium and high severity incidents were significantly increased on the owner’s property (OR = 2.38), in an off-leash park (OR = 3.20) and in the home (OR = 8.17) compared with incidents taking place in public spaces. The predicted probability of a low severity incident was highest in public spaces (0.6, 95% CI: 0.56–0.63). This was significantly higher than in all other locations and significantly higher than the probability of medium or high severity incidents in public spaces (Figure 2). The predicted probability of a medium severity incident was highest in off-leash parks (0.45, 95% CI: 0.41–0.49). This was significantly higher than the probability of a medium severity incident in a public space and significantly higher than the probability of a high severity incident in an off-leash park. The predicted probability of a high severity incident was highest in the home (0.41, 95% CI: 0.32–0.52). This was significantly higher than the probability of a high severity incident in an off-leash park, on the owner’s property or in a public space. The predicted probability for high severity incidents taking place on the owner’s property or in off-leash parks was statistically higher than those occurring in public spaces.

### 3.4. Dog Sex

**Figure 3:**
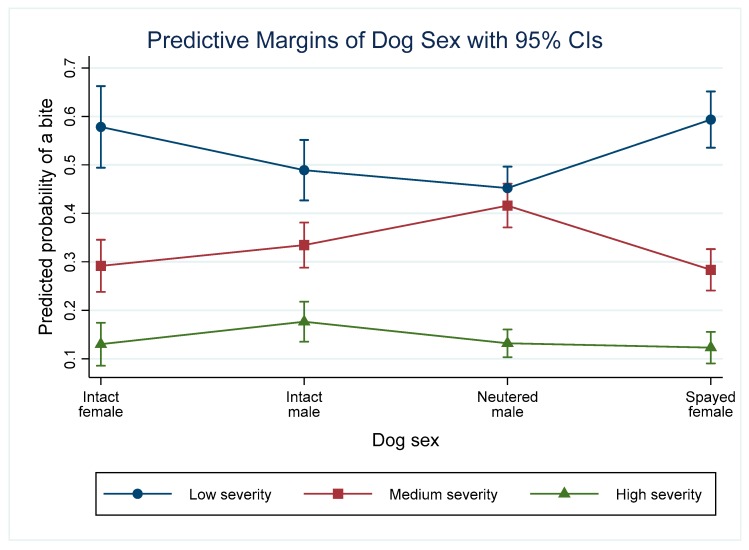
Predictive margins for the different severity incidents depending on the sex of the dog and controlling for other variables in the model.

The model indicates that the odds of a medium or high severity bite (Equation (1)) was higher in males (intact or neutered) than in intact females. Spayed females did not differ from intact females. Though there was a deviation from proportionality for neutered males, the odds of a bite among neutered males was not significantly higher for a low and medium severity bite compared with a high severity bite (Equation (2)). There were no significant differences in the predicted probabilities of high severity incidents depending on the sex of the dog (Figure 3). Spayed females had a significantly lower probability of a medium severity incident than neutered males, while neutered males had a significantly lower probability of a low severity incident than spayed females.

### 3.5. Victim Age

**Figure 4:**
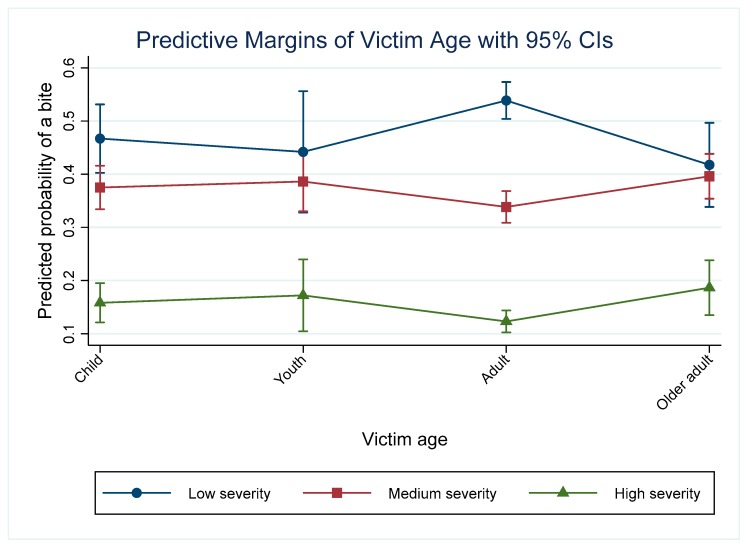
Predictive margins for the different level of dog chase/bite incidents depending on the victim age category and controlling for other variables in the model.

Amongst the 2165 incidents included in the final model, 25% of 306 high severity incidents were directed towards children, 15% towards youths, 55% towards adults and 17% towards older adults. The model indicates that the odds of a bite of any severity was higher in children (OR = 1.39), youths (OR = 1.55) and older adults (OR = 1.74) compared with adults, holding all other predictors at their baseline. The predicted probability of a high severity incident was significantly lower than that for low and medium severity incidents for all age categories (Figure 4). The predicted probability of a high severity incident was significantly higher in older adults (60+) (*p* = 0.18, 95% CI: 0.13–0.24) than in adults (20–59) (*p* = 0.12, 95% CI = 0.10–0.14) (Pairwise comparison: *p* = 0.02, 95% CI: 0.004–0.12). For medium severity incidents, there were no significant differences in the predicted probabilities between the different age categories.

### 3.6. Dog Age

**Figure 5:**
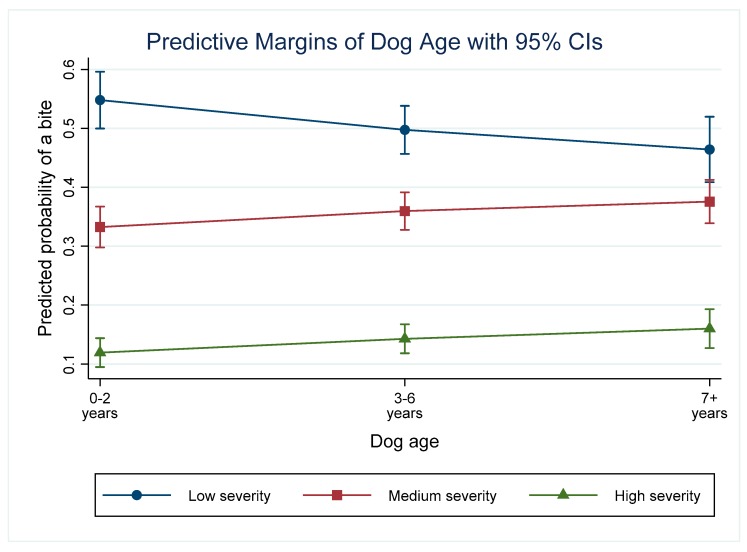
Predictive margins for the different severity incidents depending on the victim age category and controlling for other variables in the model.

The model indicates that the odds of a bite of any severity was higher in older dogs compared with dogs 0–2 years old, holding all other predictors at their baseline. When dog age is considered as the predictor of interest, the highest predicted probabilities were for low severity incidents in all age categories (Figure 5). This was significantly higher than a medium severity incident in dogs that were 0–2 or 3–6 years old. In older dogs, the predicted probability of a low severity incident was not significantly different from the probability of a medium severity incident. In all age categories, the predicted probability of a high severity incident was significantly lower than a low or medium severity incident.

### 3.7. Year

**Figure 6:**
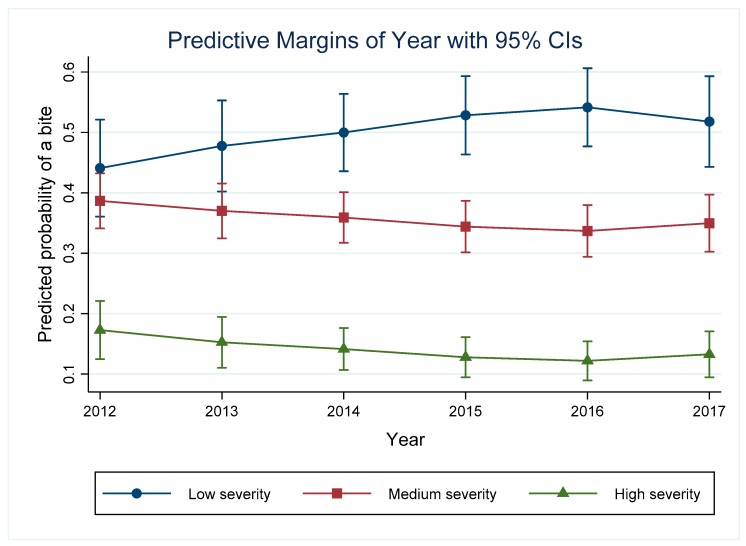
Predictive margins for the different severity incidents depending on the year and controlling for other variables in the model.

The model indicates that the odds of a low versus medium or high severity bite in 2012 was significantly different to 2017. The number of bite incidents reported in 2012 was 325. The number increased each year, to a peak of 548 in 2016. In 2017, the number of incidents reduced to 446. There has been an increasing trend in low severity incidents from 2012–2016, and a decreasing trend in medium severity incidents in the same time period. The predicted probability of a high severity incident peaked in 2012 (0.17, 95% CI: 0.12–0.22), and dropped to 0.12 (95% CI: 0.09–0.15) in 2016 (Figure 6). In 2017, there were slight increases in the predicted probabilities of both medium and high severity incidents. From 2014 onwards the predicted probabilities for the three severity groups were significantly different from each other.

### 3.8. Victim Age in Different Incident Locations

**Figure 7:**
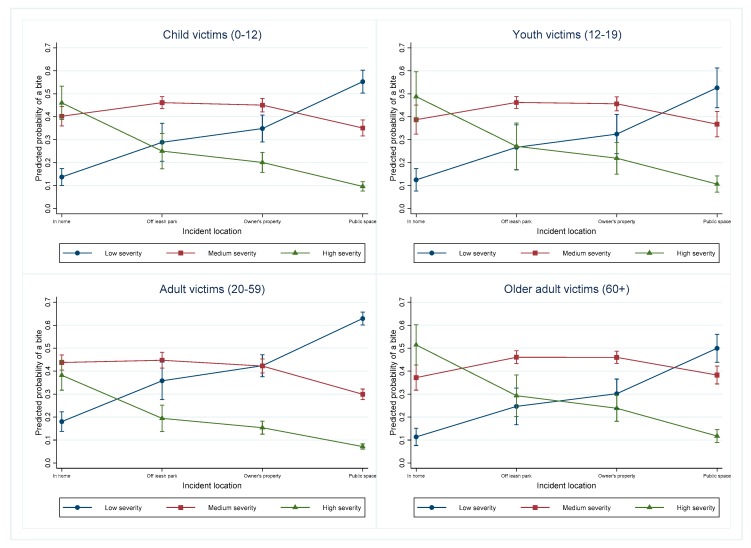
Predictive margins for the different severity incidents depending on the incident location and the victim age and controlling for other variables in the model.

The predicted probabilities of incidents at each outcome level were evaluated in the different age groups and in the different incident settings (Figure 7). The corresponding pairwise comparisons for each age group and location combination are reported (Table 4). In each age category (excluding adults), the highest predicted probabilities for a high severity bite occurred in the home. Thirty-six percent of incidents that took place in the home involved children (n = 73). Just eight (4%) involved youths, 107 (52%) involved adults, and 17 (9%) involved older adults. Child, youth and older adult victims showed very similar patterns for the different severity bites occurring in different locations. There were 13 incidents involving children, one involving youth, 76 involving adults and seven involving older adults that took place in off-leash parks. When the incident took place on the owner’s property, 68 (17%) involved children, 15 (4%) involved youth, 270 (69%) involved adults, and 36 (9%) involved older adults. When the incident took place in public, 249 (17%) involved children, 93 (6%) involved youth, 950 (64%) involved adults, and 182 (12%) involved older adults. Adults had the highest predicted probability for a low severity incident occurring in a public space.

## 4. Discussion

The aim of this work was to use an internationally recognized aggression scale tool to examine City of Calgary dog aggression related administrative data with the purpose of gaining a better understanding of the circumstances in which bites at varying severity levels occur amongst the biting dog population in a Canadian city. Utilization of such data from administrative systems for the purpose of assessing the impact of dog bites is novel, and the results of this study highlight the usefulness of utilizing such an approach when assessing the severity of dog aggression incidents in urban areas. The results of this study may be generalizable to other large cities in developed countries where administrative systems use similar methods to record dog bite severity.

The following variables were found to be significant predictors of bites of varying severity amongst the biting dog population from 2012–2017: Breed group, incident location, the age of the victim, dog sex and age, and the year of the incident. It is important to consider that the results of the model need to be evaluated in relation to the baseline values and that the predictive probabilities calculated based on the model represent scenarios where the predictor of interest is manipulated, and all other predictors are held at their values.

Three percent of dogs responsible for bites in this study were subsequently euthanized by the owner, or as a result of the investigation by The City of Calgary. This is an unfortunate outcome, considering that dog bites are generally a preventable public health issue. This raises the question of how we can better educate dog owners and the general public about safety around dogs. The results of this study indicate that such education programs should target all demographics.

### 4.1. Bite Severity

This study utilized the Dunbar bite scale as a three-level ordinal outcome of interest for predicting attributes of dog bite incidents in the City of Calgary. These data provide information on the number of dog-related aggression incidents that have taken place in the city between 2012–2017. While this is very useful information, it is likely that it is an underrepresentation of the number of incidents that actually take place, particularly incidents that may be deemed less serious. For an incident to become a part of this dataset someone needed to report it. The likelihood of this occurring may depend on the severity of the incident and the knowledge and attitude of the victim [15]. Dunbar indicates that over 99% of incidents of aggression in dogs are levels 1 and 2 [33]. In this study, level 1 and 2 incidents (low severity) were the most frequently reported (51%) followed by level 3 (medium severity) (35%). This does not reflect the percentage of low severity bites reported by Dunbar. This indicates that a large number of low severity incidents are likely not reported. The nature and magnitude of this bias on the current study are not known.

It is thought that incidents that might not result in an injury that requires medical attention (low or medium severity) may be less likely to be reported, particularly if it happens within the home. Dog owners may be less likely to report an incident where their own dog is responsible for a bite, particularly if it does not require medical treatment. Of 301 bites reported in a recent survey of 385 UK households, just 33% required any medical treatment [41]. Similarly, the majority of respondents to a self-reported survey regarding dog bites (62.3%) in the UK did not seek medical treatment, emphasizing the need to collect data on dog bites beyond medical facilities [42]. The benefit of use of administrative data such as that used in the current study is that it is likely to capture incidents that do require medical treatment, alongside less serious incidents that may not require treatment, but for which a victim or witness was concerned about the behaviour of the dog in question. While this does not capture all dog bites, it does provide more accurate data on dog bites than would be available using only medical database information, or through surveys. With an active animal control program such as that in place in Calgary, the possibility of having a dog reported to animal services promotes responsible pet ownership, encouraging pet owners to keep their dogs under control, and consider the consequences of their dogs’ actions should they be at large. That over 50% of the reported incidents are levels 1 or 2 indicates that the city’s approach to educating the public regarding dog aggression issues is working.

It is important to consider the implications of minor dog aggression incidents to physical and psychological health. A person may feel unsafe utilizing public spaces for walking or recreation where they have been the subject of a dog attack or threat. Additionally, exposure to such incidents may invoke feelings of fear [43] or symptoms of post-traumatic stress disorder [44] that may affect a persons wellbeing. Therefore, even minor bites or aggressive incidents, i.e., those classified as low severity in the current study, should be considered as part of strategies for dog bite prevention.

There is little information available regarding the severity of dog aggression incidents such as presented in the present study. However, one recent study from the UK did gather self-reported information on bite severity, with bite descriptions comparable to the Dunbar scale. In that study, there were 145 (34%) instances where a dog made one to four punctures from a single bite, with no puncture deeper than half the length of the dogs’ canine teeth, similar to a level 3 or 3.5 bite. This frequency is comparable to the percentage of level 3 outcomes reported in the present study (35.5%). This use of objective methods of describing dog bites highlights the merits of using the Dunbar scale as a tool for assessment of dog bite severity by The City of Calgary.

### 4.2. Breed Group

The results of this study indicate that dog bite severity is not dictated by the dog breed grouping. In the model, with the non-sporting breed group as the baseline, and holding all other predictors at their baseline value, sporting, and terrier breed groups had lower odds of a medium or high severity incident. However, based on the predicted probabilities calculated for each breed group, dogs classified as non-sporting were no more likely than the other breed groups to be responsible for any of the severity classifications of bites. Overall, this finding is supportive of the growing body of research that indicates that breed specific legislation is not a successful approach to dealing with dog aggression issues [45,46]. Dogs in all breed groups are capable of inflicting a bite, and the probability of a high severity bite was not significantly higher in any particular breed group.

However, the level of subjectivity involved in the classification of dogs, particularly mixed breeds, into breed groups should be considered. Breed information is reported by the owner when they license their dog. If upon visual inspection an animal services officer thinks that a dog breed has been miss-reported then the record for the animal is updated with the new breed classification. Additionally, within the Canadian Kennel Club breed group classifications, there is some variation, particularly in the non-sporting dog category. Dogs included in this category include three bull breeds, Spitz breeds, Tibetan breeds, Poodles, and others. The CKC notes on its non-sporting breed group information page that “the vast variety of breeds, purposes and personalities make it difficult, if not impossible, to assess the group’s capacity to learn” [47]. In future work, it is hoped to utilize the number of licensed dogs to more accurately analyze the risk of bites in each breed/breed group. In the current study, from 2014 onwards, for 56% of incidents reported dogs were licensed, while for 14% the license status was unknown. While we know that not all dogs in the city are registered, we also know that Calgary has a high rate of compliance with licensing bylaws. Despite high compliance, the proportion of licensed and unlicensed dogs in each breed/breed group is not likely to be equal, therefore using population-level data to analyze dog aggression issues is also fraught with difficulties.

### 4.3. Incident Location

The results of this study indicate that the severity of the incident increases when the incident takes place in the home, compared to on the owner’s property, in an off-leash park, or in a public space. Incidents taking place in off-leash parks were also shown to be a higher severity than incidents taking place in public spaces. Additionally, increased severity in the home is seen in all age categories. Medium and high severity incidents were significantly more likely to occur in the home than low severity incidents, with the reverse true when the incident was in a public space. These findings relating to increasing incident severity in the home can be considered in combination with previous research that has indicated that the majority of dog bites occur in home settings [42,48,49,50,51]. That articles reporting hospital data [48,49,50,51] indicate that bites occurring in the home are the most prevalent, indicates that bites occurring in this location are more likely to result in injury requiring medical treatment. For example, Loder (2019) [50] reported that 80% of dog bite injuries treated in emergency departments in the US occurred in the home. This is in agreement with the findings in the current study. As previously discussed, the possibility of bias needs to be considered in relation to the incident location. The vast majority of incidents took place in public (70%). Where incidents take place in a public domain and involve encounters with unfamiliar people it is more likely that they will be reported to animal services than if they occur in the home, and/or the victim is known to the dog and dog owner, particularly if the incident does not require medical attention. This is not a failing in the data, or the analysis, but rather a bias that needs to be recognized when considering the results of the study.

### 4.4. Dog Sex

Male dogs were the most commonly reported sex responsible for bites in a number of studies [42,48,52]. The results of the model indicate that male dogs are more likely to be responsible for a medium or high severity bite than intact female dogs. When this was evaluated further using margins, it was found that the probability of high severity incidents did not change significantly with the sex and spay/neuter status of the dog. Low severity incidents were significantly lower amongst neutered males than amongst spayed females. The predicted probability of a medium severity incident was significantly higher amongst neutered male dogs than amongst intact or spayed females. In the current study, neutered males accounted for the majority of incidents (39%) in the given dataset. Additionally, neutered male dogs were the most frequently reported as ‘at large’ (35%) when considered in combination with the circumstances of the incident. Therefore, further analyses are necessary to explore the relationship between dog sexes and bite severity. Future research will examine the proportion of male and female dogs based on the overall licensing information.

### 4.5. Victim Age

It was hypothesized that children would be more likely the victim of high severity incidents than older persons. This hypothesis was based on previous research indicating children are highly represented in studies utilizing hospital data examining dog bites issues [48,49,50,51]. Based on the results of the model, when holding other predictors at their baseline, children, youths and older adults all had significantly higher odds of a bite of any severity than adults. This finding is in agreement with other research [48,49,50,51]. When this was examined further using predictive probabilities, the results indicate that the risk of a high severity incident was not higher amongst children than the other age categories investigated. However, the results did indicate that the probability of a high severity incident was higher amongst older adults (60+) than adults (20–59).

The higher predicted probability of a high severity bite in older adults compared to adults in the current study is notable. In the older adult population, an association between fractures and walking dogs on leashes has recently been reported [53]. It is likely that situations leading to dog walkers suffering from fractures are the result of behavioural and/or obedience issues with the animal in question. An increased odds of admission to hospital following a dog bite injury in adults over 75 years has been reported in the US [50] Similarly, an increased risk of significant injury, defined as patients death, hospitalization, surgery, or diagnosis of fracture or amputation following a dog bite injury was reported in adults over the age of 60 in Korea [51]. These results indicate that there are substantial health concerns associated with the responsibility of dog ownership amongst the older adult populations. This may be an area where education can play a role. Utilization of courses in obedience training and education for owners in understanding dog body language should target all age groups, including older adults.

By calculating predictive probabilities it was found that the most severe bites occurred in the home in all age categories, compared to when on the owner’s property, in an off-leash park, or in a public space. This is in agreement with other international research on the subject. For example, in a questionnaire conducted in Belgium, 65/100 dog bite victims under 16 years of age were bitten in the home, while 35 were bitten in a public place [54]. However, when considering these results, the distribution of incidents amongst the age categories in the different incident settings needs to be taken into account. For example, for medium severity incidents, children, youths and older adults were poorly represented in the off-leash park setting, likely due to lower use of such resources in these age demographics. This is reflected in the wide confidence intervals associated with the predicted probabilities for all ages in this incident setting.

### 4.6. Age of the Dog

Previous research has implicated dog age as a risk factor for dog bites, with incidents in the home directed towards unfamiliar people [55], young dogs (0–5 years) reported to be responsible for more bites than older dogs [54], and adult dogs (2–10 years) responsible for more bites than other age categories [42]. These conflicting results indicate that there is no clear consensus on the effect of the dog’s age on its likelihood to bite. The results of this study indicate that older dogs (3+) had an increased odds for bites of increasing severity, however, the predicted probabilities for each bite severity level calculated indicated that there is no effect of dog age on the probability of a dog inflicting bites of different severity. In all age groups, the probability of low or medium severity incidents was significantly higher than high severity incidents, and this did not vary significantly with the age of the animal. The aim of this study was to explore risk factors for dog bite severity, as opposed to risk factors for dog bite incidence as was the aim in much of the literature published to date. Moreover, the age categories used by the city are broad (0–2, 3–6 and 7+) making it less likely to detect significant differences than if age could be treated as a continuous variable. Controlling for age in the multivariable model did not alter greatly the odds ratios compared with age in an unconditional model (For 3–6 year old dogs the OR changes from 1.28 to 1.25, and from 7+ year old dogs the OR changes from 1.55 to 1.44). This indicates that in the population being studied, while the age of the dog is associated with varying bite severity incidents, there are no significant differences within each incident level based on the age categories investigated.

### 4.7. Year of the Incident

Results indicate that while the overall number of bite incidents increased between 2012 and 2016, the number of high severity incidents did not increase, and the number of medium severity incidents decreased. There has been an increase in the predicted probability of a low severity (level 1 or 2) incident, with the majority of bites in each year being a low severity incident. This may indicate that citizens are actively reporting instances where a dog barks, growls, threatens or chases at a more frequent rate, or that such incidents are making up the majority of the reports made to animal control services. The increase in dog bite reporting may reflect the efforts of the City of Calgary Community Standards team to educate dog owners and the public regarding responsible pet ownership, and the importance of reporting all instances of dog aggression to the authorities.

### 4.8. Study Limitations

There are a number of limitations to this study. For example, the skewed distribution of a number of variables such as the circumstances of the bite, and the relationship of the victim to the dog excluded them from analysis in the multivariable model building process. It is possible that these variables will be confounders for the relationship between dog bite incidents and the actual incident setting. This should be re-evaluated when further data are available. There were a small number of incidents that involved multiple dogs, and a small number of dogs that were responsible for more than one incident. Failing to account for these types of incidents is a shortcoming of these analyses. Future analyses will aim to account for a lack of independence where multiple dogs are involved. There were a number of variables for which data were missing, including where the dog was obtained, and the years owned. Information on these variables was not collected prior to 2015. Future analyses of the dataset from 2015 forward would allow for utilization of these variables. Population-level data such as the number of licenced dogs in the City of Calgary for each year were not currently available. Future work will aim to include these data in new analyses. Census data on the human population in the city in each year would also be useful to allow for calculation of incidence rates for dog bite outcomes.

## 5. Conclusions

Dog bites are a significant public health issue that also results in negative outcomes for the animals in question, such as relinquishment or euthanasia. This article has taken a novel approach to utilizing data collected by a large urban administrative system to examine the risk factors for dog bites of varying severity. Bite prevention strategies can be informed with context-specific data regarding the type of dogs that are committing serious bites, and the situations in which these incidents occur. In utilizing the Dunbar aggression scale as the outcome of interest in this study, we can report on the probability of bites of varying severity occurring based on the dog breed group, incident setting, the victim age, and the age and sex of the dog. The results presented provide useful information that can be used to inform public policy regarding where education programs relating to dog bite prevention should be targeted. In the City of Calgary, the most severe dog bites are more likely to occur in the home and be inflicted upon all age groups, but particularly children, youths and older adults.

These results reinforce the need for education of both dog owners and the general public, including the older adult population and parents of young children. Dog owners should be aware that previous signs of aggression may predicate future incidents, and learn to read their dogs behaviour to anticipate issues before they arise. Owners of dogs responsible for low severity incidents should seek guidance from dog behaviour specialists to understand why the incident occurred and work with their dog to curtail the issue before it potentially becomes more serious.

Parent or guardian awareness and understanding of dog behaviour is critical to reduce bites [1,56]. Considering that the most severe bite incidents occur in the home, parents should supervise young children in the presence of dogs, even dogs with which the child is familiar. Outlets for education for parents and children include medical offices, schools, after-school programs, veterinary clinic visits, and local animal services officers. Similarly, the study results indicate that the older adult population is at risk of dog bites in the home, therefore, this demographic may also benefit from targeted education programs. Additional outlets for such programs may include seniors’ active living groups, and charities or organizations targeting older adult population health. Tailored education programs will help to raise awareness, with a view to reduce the incidence of this preventable public health risk, and improve animal welfare outcomes for affected dogs.

## Figures and Tables

**Table 1:** The Dr. Ian Dunbar aggression assessment scale modified by Dr. Dunbar and Calgary Animal Services in 2012.

Outcome of Interest.	Number of Incidents (2012–2017)	Assessment of the Severity of Biting Problems Based on an Objective Evaluation of Wound Pathology	
Low severity incident	1023	Level 1	Dog growls, lunges, snarls-no teeth touch skin. Mostly intimidation/threatening behaviour
	368	Level 2	Teeth touch skin but no puncture. May have red mark/minor bruise from dog’s head or snout, may have minor scratches from paws/nails. Minor surface abrasions or lacerations
Medium severity incident	955	Level 3	Punctures one to three holes, single bite. No tearing or slashes. Victim not shaken side to side. Bruising
High severity incident	111	Level 3.5	Multiple level 3 bites
	191	Level 4	Two to four holes from a single bite, typically contact/punctures from more than canines, considerable bruising. Black bruising, tears and/or slashing wounds. Dog clamped down and held and/or shook head from side to side
	65	Level 5	Multiple bites at Level 4 or above. A concerted, repeated attack causing severe injury
Not included		Level 6	Any bite resulting in death of an animal

**Table 2:** The distribution and unconditional generalized ordered logistic regression odds ratio and *p*-value for each variable considered for inclusion in the final model.

Variable	Frequency	(%)	Low Severity (Level 1 and 2)	Medium Severity (Level 3)	High Severity (Level 3.5, 4, 5)	Unconditional Generalized Ordered Logistic Regression (OR)	**^2^*p*-Value**
*^~^ Incident Location:							<0.0001
In house	227	8	22	110	95	Baseline	
Off-leash park	161	6	57	87	17	0.18	<0.001
Owner’s property	437	16	176	183	78	NPL	
Public	1888	70	1136	575	177	NPL	
* Victim age							0.0056
Child (0–12)	455	17	202	166	87	Baseline	-
Youth (13–19)	157	6	71	66	20	0.86	0.40
Adult (20–59)	1662	61	904	569	189	0.63	<0.001
Older adult (60+)	304	11	137	114	53	0.95	0.70
Unknown	135	5	77	40	18	0.60	0.008
*^~^ Dog sex:							<0.0001
Female-intact	263	10	160	71	32	Baseline	-
Male-intact	501	18	253	158	90	1.54	0.004
Male-neutered	1050	39	450	445	155	NPL	
Female-spayed	525	199	315	154	56	1.00	0.97
Unknown	374	14	213	127	34	NPL	
* Dog age:							0.0001
0–2	762	28	428	239	95	Baseline	-
3–6	1008	37	502	358	148	1.28	0.008
7+	522	19	227	217	78	1.55	<0.001
Unknown	421	16	234	141	46	0.99	0.96
* City quadrant:							0.07
Northeast	528	23	287	170	71	Baseline	-
Northwest	732	31	370	257	105	1.15	0.21
Southeast	537	23	249	198	90	1.37	0.008
Southwest	528	23	257	204	67	1.18	0.16
*^~^ Year:							<0.0001
2012	325	12	134	147	44	NPL	
2013	360	13	165	142	53	1.17	0.25
2014	511	19	260	182	69	0.98	0.87
2015	523	19	280	186	57	0.86	0.23
2016	548	20	320	158	70	0.75	0.02
2017	446	16	232	140	74	Baseline	-
* Month:							0.16
January	160	6	83	60	17	Baseline	-
February	135	5	67	44	24	1.22	0.37
March	187	7	87	73	27	1.26	0.25
April	254	9	147	74	33	0.85	0.41
May	259	9	124	101	34	1.18	0.38
June	365	13	201	118	46	0.93	0.70
July	295	11	142	111	42	1.2	0.33
August	290	11	142	107	41	1.17	0.41
September	255	9	147	79	29	0.84	0.36
October	239	9	117	90	32	1.15	0.47
November	145	5	71	56	18	1.13	0.57
December	129	5	63	42	24	1.27	0.29
* Prior aggression							0.07
No	1817	67	932	885		Baseline	-
Yes	418	15	197	221		1.18	0.12
Unknown	478	18	262	216		0.87	0.17
^^^ Victim Gender							0.41
Male	1349	50	675	498	176	1.06	041
Female	1364	50	716	457	191	Baseline	-
*^~^ Breed group:							0.006
Herding	630	23	315	234	81	0.83	0.18
Hound	66	2	31	32	3	0.79	0.35
Non-sporting	251	9	111	107	33	Baseline	
Sporting	435	16	254	133	48	0.60	0.001
Terrier	618	23	334	189	95	NPL	
Toy	155	6	72	72	11	0.83	0.35
Working	558	21	274	188	96	NPL	
^^^ Circumstances:							
At large	1525	56	1011	385	129		
Contained	111	4	30	43	38		
Dog fight	82	3	13	41	28		
Guard dog	2	0	0	1	1		
Multi-dog	153	6	120	25	8		
On leash	243	9	78	136	29		
Other	444	16	115	242	87		
Provoked	81	3	7	48	26		
Tethered	65	2	14	30	21		
Unknown	7	0	3	4	0		
^1^^ Ward							
1	149	5.5	72	59	18		
2	145	5	71	58	16		
3	166	6	86	60	20		
4	179	7	83	67	29		
5	244	9	118	88	38		
6	113	4	59	44	10		
7	117	4	55	42	20		
8	100	4	46	38	16		
9	241	9	111	89	41		
10	259	10	120	90	49		
11	154	6	86	55	13		
12	179	7	107	50	22		
13	130	5	83	27	20		
14	149	5.5	66	62	21		
Out of town/Unknown	388	14	228	126	34		
^^^ Controlled by:							
Child	68	2.5	23	30	15		
Family	264	10	109	121	34		
Friend	128	5	64	51	13		
None	881	32	598	198	85		
Owner	1318	46	567	539	212		
Professional	31	1	11	13	7		
Unknown	23	1	19	3	1		
^^^ Relationship with dog:							
Family	122	4.5	12	53	57		
Guest	88	3	11	37	40		
Neighbour	722	27	458	194	70		
None	1504	55	769	564	171		
Service provider	277	10	141	107	29		
^^1^ Where obtained:							
Breeder	289	11	153	103	33		
Other	474	17	225	164	85		
Rescue/Shelter	222	8	104	85	33		
Unknown	1728	64	909	603	216		
^^1^ Vaccination status:							
Not vaccinated	469	17	256	149	64		
Vaccinated	1144	42	510	456	178		
Unknown	1100	41	626	350	125		
^^1^ Licence status							
No licence	611	23	306	215	90		
Licence	1128	42	607	362	159		
Unknown	974	36	478	378	118		
^^1^ Years owned the dog:							
Less than 1 year	187	7	100	54	33		
1–3 years	419	15	216	142	61		
3–5 years	207	8	111	74	22		
5+ years	212	8	94	86	32		
Unknown	1688	62	870	599	219		

* Variables included in the model building process; ^^^ Variables not included in the model building process; ^1^ Data not collected in all years, therefore where data was not available the outcome is unknown. Due to the large number of unknowns in these categories they were not further assessed; ^2^ An overall *p*-value for each categorical variable assessed in an unconditional association is provided, as well as the *p*-value for each category within the predictor. For the unconditional associations, an odds ratio and *p*-value are not provided for categories within a predictor if the category did not meet the assumptions for parallel lines (NPL). Where that predictor was subsequently significant in the final model, the associated odds ratios and *p*-values are reported (Table 3); ^~^ NPL = non-parallel lines. Model assumptions for parallel lines were not met.

**Table 3:** Multivariable generalized ordered logistic regression model.

Multivariable Generalized Ordered Logistic Regression (n = 2165)	Low versus Medium and High (Equation (1))			Low and Medium versus High (Equation (2))			* Gamma (Deviations from Proportionality)	
	OR	*p*-Value	95% CI	OR	*p*-Value	95% CI	OR	*p*-Value
Breed Group (baseline is non-sporting)								
Herding	0.89	0.456	0.65–1.21					
Hound	0.68	0.184	0.38–1.20					
Sporting	0.69	0.032	0.49–0.97					
Terrier	0.71	0.041	0.51–0.99	1.10	0.630	0.75–1.61	1.55	0.003
Toy	0.79	0.277	0.52–1.20					
Working	0.94	0.689	0.68–1.29					
Incident location (baseline is public space)								
In home	8.17	<0.001	6.08–10.98					
Off-leash park	3.20	<0.001	2.18–4.68					
Owner’s property	2.38	<0.001	1.91–2.97					
Dog Sex (baseline is intact female)								
Intact male	1.50	0.01	1.09–2.06					
Neutered male	1.77	<0.001	1.31–2.40	1.02	0.921	0.71–1.46	0.57	<0.001
Spayed female	0.93	0.67	0.67–1.29					
Victim age (baseline is adult)								
Child	1.39	0.004	1.11–1.73					
Youth	1.55	0.020	1.07–2.25					
Older adult	1.74	<0.001	1.33–2.27					
Dog age (baseline is 0–2)								
3–6	1.26	0.026	1.03–1.54					
7+	1.46	0.002	1.16–1.86					
Year (baseline is 2017)								
2012	1.42	0.034	1.03–1.97					
2013	1.20	0.253	0.88–1.65					
2014	1.09	0.579	0.81–1.46					
2015	0.95	0.750	0.71–1.28					
2016	0.90	0.473	0.67–1.21					
Constant	0.40	<0.001	0.25–0.62	0.06	<0.001	0.04–0.10		

* Gammas represents the difference in coefficients (logit scale) from Equation (2) − Equation (1). For example: For the terrier breed group Equation (1): log (0.71) = −0.34, Equation (2): log (1.10) = 0.09, Gamma: log (1.55) = 0.438. To calculate Gamma: = 0.09 − (−0.34) = 0.435.

**Table 4:** Pairwise comparisons for each victim age group at each incident setting.

Severity and Location of the Incident:	Child Victim	Youth Victim	Adult Victim	Older Adult Victim
Low severity—In home	AB	AB	A	AB
Low severity—Off-leash park	CDE	ABCDE	BCD	CDE
Low severity—Owner’s property	CDEF	CDEF	D	DE H
Low severity—Public space	G	G		FG
Medium severity—In home	DEF	CDEFG	D	DE HI
Medium severity—Off-leash park	FG	FG	D	G
Medium severity—Owner’s property	FG	E	D	I
Medium severity—Public space	DE	DEFG	BC	EF H
High severity—In home	EFG	E	CD	HI
High severity—Off-leash park	BCD	BCD FG	AB	B DEFG
High severity—Owner’s property	BC	BC	A	B D
High severity—Public space	A	A		A C

Margins sharing a letter in the group label are not significantly different at the 5% level.
